# Cardiovascular disease, risk factors, and health behaviors among cancer survivors and spouses: A MEPS Study

**DOI:** 10.1002/cam4.3336

**Published:** 2020-08-04

**Authors:** Lixin Song, Ting Guan, Peiran Guo, Fengyu Song, Courtney Van Houtven, Xianming Tan, Thomas C. Keyserling

**Affiliations:** ^1^ School of Nursing University of North Carolina at Chapel Hill (UNC‐CH) Chapel Hill NC USA; ^2^ Lineberger Comprehensive Cancer Center UNC‐CH Chapel Hill NC USA; ^3^ School of Social Work UNC‐CH Chapel Hill NC USA; ^4^ General Education West Coast University Anaheim CA USA; ^5^ Center of Innovation to Accelerate Discovery and Practice Transformation (ADAPT) Durham Veterans Affairs Health Care System Durham NC USA; ^6^ Department of Population Health Sciences Duke University School of Medicine Durham NC USA; ^7^ Margolis Center for Health Policy Duke University Durham NC USA; ^8^ Gillings School of Global Public Health UNC‐CH Chapel Hill NC USA; ^9^ School of Medicine UNC‐CH Chapel Hill NC USA

**Keywords:** cancer, cardiovascular disease (CVD), family, health behavior, Medical Expenditure Panel Survey (MEPS), quality of life

## Abstract

**Purpose:**

The purpose of this study was to examine the prevalences of CVD, CVD risk factors. and health behaviors among cancer survivor‐spouse dyads, assess how these prevalences differ by role (survivor vs spouse) and gender, and report congruences in health behaviors between survivors and their spouses.

**Methods:**

We identified 1026 survivor‐spouse dyads from the 2010‐2015 Medical Expenditure Panel Survey. We used weighted multivariable logistic and linear regressions to analyze the data related to CVD, CVD risk factors, and health behaviors.

**Results:**

Survivors and spouses reported high prevalences of CVD and CVD risk factors but low engagement in healthy behaviors, including non‐smoking, physical activity, and maintaining a healthy weight (proxy for healthy diet). Gender and role differences were significantly related to the prevalence of CVD, CVD risk factors, and health behaviors among survivors and spouses. From 39% to 88% of survivors and spouses were congruent in their current smoking status, physical activity engagement/disengagement, and BMI.

**Conclusion:**

Cancer survivors and spouses have high rates of CVD and CVD risk factors and poor engagement in healthful lifestyle behaviors. A high proportion of survivors and spouses were congruent in their current smoking status, physical activity engagement/disengagement, and BMI. Effective lifestyle interventions are needed for this high‐risk population. Couple‐focused interventions may be well‐suited for these dyads and warrant further study.

**Implications for Cancer Survivors:**

Both cancer survivors and their spouses need to be non‐moking, more physically active, and maintain normal BMI in order to reduce their high risk of CVD and CVD risk factors.

## Graphical abstract

Cancer survivors and spouses have high rates of CVD and CVD risk factors and poor engagement in healthy lifestyle behaviors. High proportion of the survivors and spouses are congruent in their current smoking status, physical activity engagement/disengagement, and BMI. Effective lifestyle interventions are needed for this high risk population. Couple‐focused interventions may be particularly well‐suited for these dyads and warrants further study.

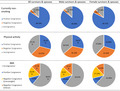

## 1 INTRODUCTION

The rapidly growing number of long‐term and older cancer survivors has increased concern about late cancer treatment‐related adverse effects and health outcomes attributed to common comorbidities among cancer survivors. Of particular concern is cardiovascular disease (CVD; eg heart attack, stroke) and major CVD risk factors (eg hypertension, high cholesterol, and diabetes)^1^ as CVD has become the leading cause of noncancer mortality among cancer survivors.^2^, ^3^ While cancer survivors in general have a higher risk of CVD compared with the general population,^4^ male cancer survivors have a higher prevalence of CVD than their female counterparts.^5^ Because the risk and severity of CVD are associated with health behaviors (eg smoking, diet, and exercise),^6^, ^7^, ^8^, ^9^, ^10^ the American Cancer Society (ACS) recommends that cancer survivors avoid smoking, engage in regular physical activity, maintain a healthy weight, and consume at least five servings of fruits and vegetables each day.^11^, ^12^ However, many cancer survivors do not follow these recommendations.^13^, ^14^ Research has also found gender differences in health behaviors among cancer survivors. Compared to male survivors, females maintain a healthier diet but engage in lower rates of physical activities.^15^, ^16^

For cancer survivors in an intimate relationship, the impact of cancer extends to their spouses,^17^, ^18^ who are often the primary source of support and caregiver of cancer patients.^10^ Survivors’ spouses experience persistent psychological distress and are also at increased risk for CVD.^19^ A study using a large cancer caregiver sample (N = 774 with 65.1% spouses) found that the most commonly reported medical morbidities among caregivers were heart disease and CVD risk factors, such as hypertension and high cholesterol.^20^ Unhealthy behaviors, eg tobacco smoking and physical inactivity, are also common among cancer caregivers.^21^ With regard to health behaviors of survivors and their spouses, interdependence theory posits that the marriage and interactions between couple members may facilitate health behavior changes, which can be either health‐compromising or health‐enhancing.^22^ Cancer survivors and spouses often have similar or congruent health behaviors, including physical activity and diet,^23^ and spouses are strongly influenced by survivors in terms of health behavior change.^24^

Given the growing burden of CVD and CVD risk factors in cancer survivors and spouses, as well as the interdependent influence of health behaviors between partners, attention to cardiovascular health and health behaviors among couples coping with cancer has become increasingly important. Although previous research has investigated the prevalence of CVD, CVD risk factors, and health behaviors among cancer survivors and spouses, most studies have assessed survivors and spouses separately. The few studies that have examined survivors and spouses as dyads used small samples of patients with specific types of cancer.^23^ Furthermore, little research has systematically examined (a) role (survivor vs spouse) and gender differences related to CVD and CVD risk factor prevalence and (b) congruence of health behaviors between cancer survivors and their spouses.

This study used a sample from a set of large‐scale surveys of cancer survivors and their spouses across the US to examine (a) the prevalence of CVD, CVD risk factors, and health behaviors (smoking, physical activity and healthy diet); (b) the role and gender differences in prevalence of CVD, CVD risk factors, and health behaviors; and (c) the congruence (positive and negative) in health behaviors between survivors and their spouses. Based on prior research and interdependence theory, we hypothesized that most cancer survivors and spouses would have high levels of congruent health behaviors.

## 2 METHODS

### 2.1 Study population

We analyzed the publicly available deidentified data from the Medical Expenditure Panel Survey (MEPS) (https://www.meps.ahrq.gov/mepsweb/) between 2010 and 2015. MEPS is a set of large‐scale surveys of health care utilization and expenditures of noninstitutionalized individuals, their families, medical providers, and employers across the United States. Information about MEPS methodology has been published previously.^25^ Adult cancer survivors (aged ≥ 18 years) were identified using respondents’ response to the question ‘‘Have you ever been told by a doctor or other health professional that you had cancer or a malignancy of any kind?’’ Respondents were deemed to be cancer survivors and eligible for this study if they reported to have been diagnosed with at least one type of the following 10 types of cancers, ie breast, prostate, lung, colon, bladder, uterine corpus, melanoma, non‐Hodgkin lymphoma, thyroid, and kidney ‐ the most common types of cancers in the United States from 2010 to 2015. Spouses were linked to the survivors by a spousal identifier if survivors reported being married.

### 2.2 Measurement

According to the American Heart Association^26^ and MEPS,^27^ CVD includes coronary heart disease, stroke, angina, heart attack, and other heart disease, and major CVD risk factors include hypertension, high cholesterol, and diabetes. Adult respondents were asked whether they had ever been diagnosed as having one or more of these conditions.

The health behaviors reported, including smoking, physical activity, and diet intake, were based on the US Preventive Services Task Force (A and B recommendations).^28^ Smoking status was assessed using the question “currently smoke” answered by yes or no. Physical activity was measured using the question “currently spend half hour or more in moderate to vigorous physical activity at least five times a week” answered by yes or no. As MEPS did not include questions on dietary intake, we used Body Mass Index (BMI) as a crude proxy of diet quality.

Covariates of this study included participant age, gender, race/ethnicity, education, insurance coverage, family income, census region, and cancer type, which were obtained from the MEPS household component. Because the study variables were not asked in each round of the surveys, we used the CVD, CVD risks, and health behaviors noted in the earliest round (eg currently smoke was measured in rounds 2 and 4, the variable in round 2 was used) and used covariates that were measured in the same round or the nearest round of outcomes.

### 2.3 Statistical analyses

To examine the prevalence of CVD, CVD risk factors, and health behaviors, we calculated weighted percentages (SAS Proc surveyfreq) of dyads’ (survivors and spouses) self‐reported diagnosis of CVD, CVD risk factors, and health behaviors. We utilized dichotomous variables to indicate whether cancer survivors and spouses met recommended guidelines for smoking and physical activity. Based on the criteria from the Centers for Disease Control and Prevention,^29^ cancer survivors and spouses were categorized as underweight (BMI < 18.5), normal (BMI = 18.5‐24.9), overweight (BMI = 25.0‐29.9) and obese (BMI ≥ 30.0).

To examine the role and gender differences in the prevalence of CVD, CVD risk factors, and health behaviors, we used weighted multivariable logistic regressions (SAS Proc surveylogistic) that modeled the effects of gender, role (survivor vs spouse) and the gender*role interaction on the categorical outcomes while controlling for covariates (ie, age, race, education level, insurance status, poverty status). This weighted regression approach accounted for the fact that MEPS has a complex sample design and also accounted for the dependence of outcomes of couples from the same family (families are considered as clusters in the MEPS survey).^30^ When the interaction effect was not significant, the models were refitted without the interaction term to obtain the parsimonious models. The same process for continuous outcomes was conducted using weighted linear regression (SAS Proc surveyreg). We used personal weights (ie, reflecting adjustments for survey nonresponse from the panel survey) in all regression analyses.

To examine the congruence in health behaviors between cancer survivors and spouses, we calculated the weighted proportion of couples that were positively congruent, negatively congruent, and incongruent for health behavior guidelines. Couples were considered positively congruent if both met the recommended health behaviors guidelines outlined by the US Preventive Services Task Force (A and B recommendations)^28^; negatively congruent if neither met the guidelines; or incongruent if one met the guidelines for each health behavior and the other did not. We used family weights in congruence analyses because congruence in health behaviors is a family level outcome. We examined the congruence among all survivors and spouses, and also based on the gender of survivors. Missing data were not a severe issue in this study because the number of variables with missing values was small and missing rates were low (ranging between 0% and 9.16%). We followed the pairwise deletion approach to include as many as possible samples in each analysis.^31^ This caused slight variation in sample sizes across analyses of different outcomes.

## 3 RESULTS

We identified 1026 cancer survivor‐spouse dyads (Table 1). The majority of survivors were white (89.28%) and equal numbers were male and female. The most commonly diagnosed cancers were breast (28.16%), prostate (25.72%), melanoma (16%), and colon (7.52%). The majority of spouses were white (88.71%) and female (51.07%). The mean ages were 62.2 and 61.2 years for survivors and spouses respectively.

**TABLE 1:** Sample characteristics

Characteristics	Survivors		Spouses		
	N	%	N	%	
Gender					
Female	513	49.75%	514	51.07%	
Male	513	50.25%	512	48.93%	
Race					
White	813	89.28%	810	88.71%	
Blacks	145	6.73%	146	6.85%	
Others	68	3.99%	70	4.44%	
Hispanic					
Yes	155	6.75%	164	7.07%	
Family income^b^					
Poor and near poor	133	9.21%	136	9.43%	
Low	132	10.59%	131	10.71%	
Middle	310	28.06%	307	28.54%	
High	451	52.14%	452	51.32%	
Education					
≤8th grade	87	5.10%	75	4.29%	
9th‐12th grade	76	4.75%	76	5.16%	
GED or high school degree	301	28.55%	326	31.91%	
Associate's degree or <4‐year college degree	261	25.84%	241	24.51%	
4‐year college degree, bachelor's degree	163	18.44%	183	17.99%	
Master's, doctorate, or professional degree	138	17.32%	125	16.16%	
Insurance coverage					
Private	691	73.47%	705	74.40%	
Public	291	23.11%	248	21.04%	
Uninsured	44	3.42%	73	4.56%	
Census region					
Northeast	172	16.46%	172	16.02%	
Midwest	210	22.27%	210	22.69%	
South	401	40.39%	401	40.04%	
West	243	20.89%	243	21.25%	
Cancer type^a^					
Breast	275	28.16%	NA		
Prostate	280	25.72%			
Melanoma	134	16.00%			
Colorectal	92	7.52%			
Uterus	77	7.28%			
Non‐Hodgkin lymphoma	51	6.42%			
Lung	51	4.11%			
Bladder	39	3.88%			
Kidney	22	6.30%			
Thyroid	40	6.20%			
	Mean	SD	Mean		SD
Age	62.20	0.50	61.17		0.51

Note

Percentages reflect weighting to account for population and family variances in the studies examined.

^a^ Patients could have more than one type of cancer.

^b^ Family income in MEPS was derived by constructing person‐level total income comprising annual earnings from various of sources, described as a percentage of poverty that was computed by dividing all person‐level total income of a family (family income) by the applicable poverty line (based on family size and composition), classified into one of five poverty categories: negative or poor (less than 100%), near poor (100% to less than 125%), low income (125% to less than 200%), middle income (200% to less than 400%), and high income (greater than or equal to 400%). (See MEPS documentation for details).

### 3.1 Prevalence of CVD, CVD risk factors, and health behaviors

**FIGURE 1:**
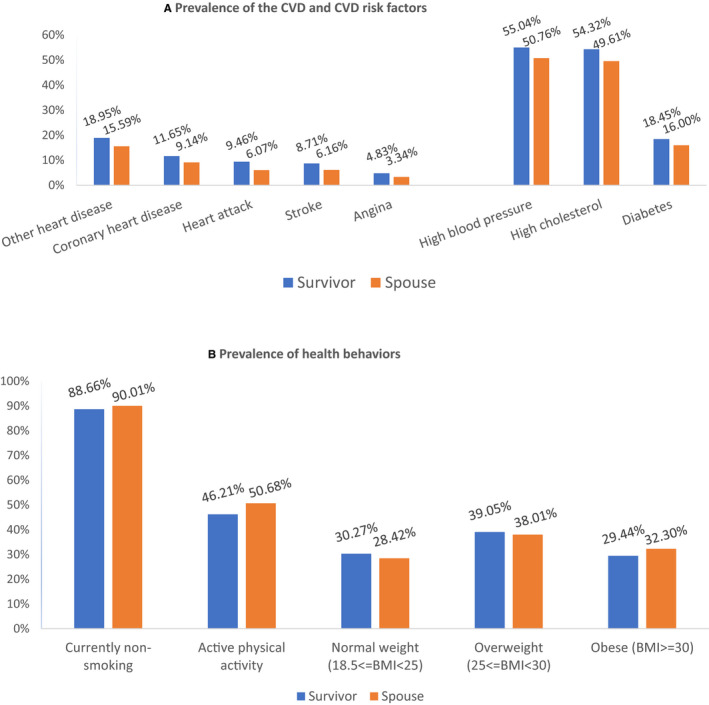
A, Prevalence of the CVD, CVD risk factors, and (B) health behaviors

The most common types of CVD and risk factors were similar among survivors and spouses (Figure 1A) including high blood pressure, high cholesterol, other heart disease (ie any other kind of heart disease or condition excluding coronary heart disease, angina, or heart attack) and diabetes. The prevalence of health behaviors was also similar in survivors and spouses (Figure 1B).

### 3.2 Role and gender differences in prevalence of CVD, CVD risk factors, and health behaviors

In the full model (Table 2), the odds of males (averaged across roles) having coronary heart disease, heart attack, high blood pressure, high cholesterol, and diabetes were, respectively, 1.46 (*P* < .001), 1.45 (*P* < .01), 1.13 (*P* < .05), 1.19 (*P* < .01), and 1.23 (*P* < .01) times as high as the odds for females, The interaction effect of role and gender on the prevalence of angina was positive and significant (*P* < .01), suggesting that the gender effect (male vs female) was significantly stronger among patients than among spouses; the odds of reporting angina were the highest among male survivors but lowest among female spouses.

**TABLE 2:** Associations between role, gender, and prevalence of CVD, CVD risk factors, and health behaviors as assessed in multivariable model^a^

	Covariates	*β*	Wald	Odds ratio	95% Confidence interval	
CVD & CVD risk factors						
Coronary heart disease	Role	0.06	0.28	1.06	0.85	1.32
	Gender	0.38	12.57***	1.46	1.18	1.80
	Role × gender	0.0025	0	1.00	0.83	1.21
Angina	Role	0.09	0.38	1.09	0.83	1.44
	Gender	0.22	2.37	1.25	0.94	1.65
	Role × gender	0.38	6.87**	1.46	1.10	1.93
Heart attack	Role	0.14	1.77	1.15	0.93	1.43
	Gender	0.37	10.8**	1.45	1.16	1.81
	Role × gender	0.13	1.15	1.14	0.90	1.42
Other heart disease	Role	0.1	1.7	1.11	0.95	1.29
	Gender	−0.009	0.02	0.99	0.86	1.14
	Role × gender	0.06	0.57	1.06	0.91	1.23
Stroke	Role	0.19	2.75	1.21	0.97	1.50
	Gender	−0.04	0.13	0.96	0.76	1.21
	Role × gender	0.2	3.17	1.22	0.98	1.53
High blood pressure	Role	0.09	3.21	1.09	0.99	1.21
	Gender	0.12	4.32*	1.13	1.01	1.27
	Role × gender	0.09	2.35	1.09	0.98	1.22
High cholesterol	Role	0.09	2.55	1.09	0.98	1.23
	Gender	0.17	8.83**	1.19	1.06	1.32
	Role × gender	0.09	2.28	1.09	0.97	1.24
Diabetes	Role	0.06	0.71	1.06	0.92	1.22
	Gender	0.21	6.98**	1.23	1.05	1.43
	Role × gender	0.1	1.39	1.11	0.94	1.30
Health behaviors						
Currently nonsmoking	Role	−0.12	2.36	0.89	0.76	1.03
	Gender	−0.21	8.4**	0.81	0.71	0.94
	Role × gender	−0.02	0.05	0.98	0.81	1.19
Physical activity	Role	−0.09	3.77	0.91	0.84	1.00
	Gender	0.19	15.01***	1.21	1.10	1.33
	Role × gender	−0.04	0.46	0.96	0.86	1.08
BMI^b^	Role	−0.23	5.21*			
	Gender	0.5	201.37***			
	Role × gender	0.73	12.35**			

^a^ In models, referent for role is spouse and for gender is female. Covariates include age, race, education level, insurance status, poverty status, and Role*Gender interaction. When Role*Gender was insignificant, we refitted the models without this interaction term. However, results were very similar to the full models which are reported in this table.

^b^ The odds ratio of BMI and 95% confidence interval of the odds ratio are not reported because BMI is a continuous outcome.

* *P* ≤ .05.

** *P* ≤ .01.

*** *P* ≤ .001.

Regarding health behaviors, the odds of males (averaged across roles) currently non‐smoking was 0.81 (*P* < .01) and the odds of males engaging in physical activity was 1.21 (*P* < .001) compard to females. The interaction of role and gender effects on BMI was positive and significant (*P* < .01), indicating that the gender effect (male vs female) was significantly stronger among patients than among spouses; the male survivors had the highest BMI and female survivors had the lowest BMI. The results of the parsimonious model (Appendix: 1) were very similar to the full model results, thus they were not reported.

### 3.3 Congruence in health behaviors between survivors and their spouses

**FIGURE 2:**
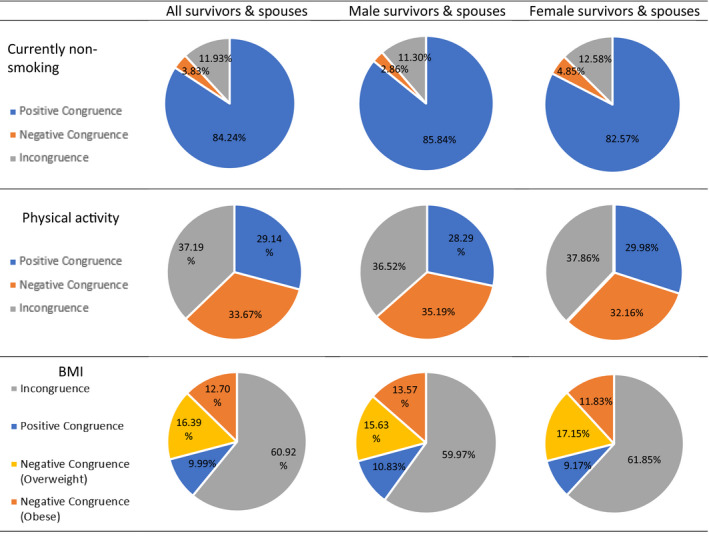
Congruence of health behaviors between cancer survivors and their spouses

Regarding smoking (Figure 2), 88.07% (n = 746) of all couples were congruent, including 84.24% positively congruent (neither partner was smoking) and 3.83% negatively congruent couples (ie both survivors and spouses were smoking). For physical activity (Figure 2), 62.81% (n = 590) of cancer survivors and their spouses were congruent, 29.14% were positively congruent (both partners physically active) and 33.67% were negatively congruent (neither physically active). For BMI (Figure 2), 39.08% (n = 389) of couples were congruent, including 9.99% positively congruent (both partners’ BMI were within normal range). Among 29.09% (n = 286) of the couples who had negatively congruent BMI, 16.39% were both overweight and 12.70% were both obese. The congruence results of these health behaviors among male and female cancer survivors and their spouses were similar to those of all survivors and spouses (see Figure 2).

## 4 DISCUSSION

In this study using the data from a set of large surveys across the US, we found high prevalence of CVD and CVD risk factors (eg high blood pressure, high cholesterol, and diabetes) among cancer survivors and spouses, and a significant proportion of this population did not report engaging in healthy behaviors (ie nonsmoking, regular physical activity, and maintaining healthy weight). In addition, we identified gender and role differences in the prevalence of CVD, CVD risk factors, and health behaviors among survivors and spouses. Consistent with existing evidence of substantial gender differences in the prevalence of CVD in the general population,^32^ we found gender differences in the prevalence of CVD and CVD risk factors among cancer survivors and spouses. Importantly, we found that 39% to 88% of the survivors and spouses were congruent (positive or negative) in their smoking status, physical activity and BMI. The results of this study complement and expand the existing evidence on CVD risk and lifestyle behaviors among healthy couples,^33^ cancer patients in a national inpatient sample database,^34^ and cancer caregivers.^20^

CVD and CVD risk factors are common among cancer survivors and their spouses.^4^ In fact, CVD has become the leading cause of death in cancer survivors.^3^ Cancer survivors may experience adverse CVD events related to their cancer treatment (eg chemotherapies and radiation therapies) or as a result of progression of underlying CVD and CVD risk factors.^35^, ^36^ Similarly, spousal caregivers are at an increased risk for CVD and CVD risk factors (eg stroke, hypertension).^37^, ^38^, ^39^ Adding to this literature, our study examined the prevalences of CVD and CVD risk factors among survivors and their spouses, as well as gender and role differences in prevalence. Although risk is high for both members of this dyad, we observed (a) that the odds of males reporting CVD and CVD risk factors (such as coronary heart disease, heart attack, high blood pressure, high cholesterol, and diabetes) were significantly higher than those of females and (b) a gender and role interaction effect on angina (ie male survivors had the highest odds while female spouses had the lowest odds of reporting angina). The collective evidence suggests that male survivors and spouse caregivers may need special support to reduce their CVD risks and promote health.

Lack of physical activity, smoking, and being overweight or obese have been associated with an increased risk of CVD and CVD risk factors,^10^, ^40^ cancer recurrence,^41^, ^42^ and mortality among cancer survivors.^43^, ^44^, ^45^ It is concerning that survivors and spouses in this sample across the US had unhealthy behaviors (ie smoking, physical inactivity, and overweight or obese). Approximately 10% of the survivors and spouses were smokers, which is lower than the general population in the United States (19.3%) ^46^; while about half of the survivors and spouses met the physical activity guideline recommended by ACS,^11^ which is equivalent to the American adults (47.1%‐50.0%) ^47^; and 68%‐70% were overweight or obese, which is slightly lower than the American adults (>70%).^29^ Consistent with interdependence theory, which suggests marriage and interactions between couple members may facilitate health behavior change,^22^ we found moderate to high congruence in health behaviors between cancer survivors and spouses, including high levels of positive congruence in their nonsmoking status, physical activity engagement/disengagement, and overweight and obese BMI. Our work extends the large body of evidence that has shown the congruence of health behaviors within healthy couples (eg dietary intake, smoking and alcohol consumption)^48^ to cancer survivors and their spouses. Couples often share a lifestyle, have a common living environment, pool resources, and cope with common stressors^48^ (such as cancer diagnosis and/or treatment). Shared major and minor life events contribute to behavioral convergence, contributing to couples' similar health and health behaviors and tendency to converge over time.^49^

Our findings, in conjunction with the literature on health risk for cancer survivors and their spouses, underscore the need to develop effective lifestyle interventions for this high‐risk population. Because both survivors and spouses are at high risk for lifestyle‐related morbidity and mortality, couples‐based therapy may be an efficient and effective modality of care and should be a high priority area for future research. In support of a focus on couples‐based interventions, researchers have documented strong spousal associations in smoking, exercise, alcohol consumption, diet, and obesity.^49^, ^50^, ^51^ Therefore, involving partners in behavior change interventions may help improve the outcomes (nonsmoking, more physical activity engagement, and normal BMI). Research has also shown that men and women are more likely to make a positive health behavior change if their partner does so, and with a stronger effect than if the partner had been consistently healthy in that domain.^51^ Therefore, interventions that are targeted toward cancer survivors but also tailored to the spouse's needs may be most effective at helping cancer survivors and spouses make positive health behavior change and maintain a healthful lifestyle over time.

Compared to the general population, cancer survivors and spouses more commonly receive certain preventive health care services (eg serum cholesterol testing, routine physical check‐ups and flu vaccination) potentially due to their relatively frequent contact with medical providers.^4^ Given this frequent contact, innovative interventions should be developed and evaluated for this clinical context to grasp the teachable moments in support of improving lifestyle behaviors. In addition to the clinical context, cancer survivors and spouses should also be encouraged to engage in community‐based programs that promote a healthful lifestyle.

The study has the following limitations. As several studies using MEPS have indicated,^52^, ^53^, ^54^ publically available MEPS data do not include the time of cancer diagnosis, stage, and treatments; therefore, we could not examine whether CVD, risk factors, and health behaviors in cancer survivors and spouses change over time. Neither were we able to discern the effects of cancer stage and related treatment. However, according to the American Cancer Society Nutrition and Physical Activity Guidelines for Cancer Survivors, all survivors, regardless of their type of cancer and stage, are encouraged to maintain healthy behaviors across the continuum of survivorship.^11^ MEPS uses the self‐report assessments for CVD, risk factors, and health behaviors, which are typically inferior to objective indicators. MEPS also has limited dietary data. In addition, this study combined data across different types of cancer, which may have masked important heterogeneity in CVD and CVD risk factors. However, previous population‐based research has found little variability in health behavior patterns (physical inactivity, diet, and being obese) except for smoking.^13^ Future research needs to include cancer‐specific information in modeling and objective data as well. In addition, we did not report on the predictors of congruence in health behaviors among couples, a topic we will address in a future report.

Nonetheless, this study contributes to the limited literature describing the prevalence of CVD, CVD risk factors, and health behaviors in couples among cancer survivors and their spouses. Using the data from a set of large surveys across the United States, our findings suggest the need for developing effective, couple‐focused and role/gender‐tailored interventions to promote health behaviors and improve cardiovascular health in this population.

## CONFLICT OF INTEREST

The authors declare that they have no conflict of interest.

## AUTHOR CONTRIBUTIONS

Conceptualization: LS, FS, CVH. Methodology: LS, PG, XMT. Data analyses: LS, PG, XMT. Writing (manuscript preparation, visualization): LS, TG, PG, FS, XMT. Writing (review and editing): LS, FS, CVH, XMT, TCK. Project administration: LS, TG, PG. Supervision: LS.

## COMPLIANCE WITH ETHICAL STANDARDS

This study involved secondary database analysis of a publicly available database, and hence, formal consent is not required.

## Data Availability

Data used in this investigator are publically available at https://www.meps.ahrq.gov/mepsweb/

## Appendix 1. Associations between role, gender, and prevalence of CVD, CVD risk factors, and health behaviors as assessed in parsimonious multivariable model.

**Table:**

CVD & CVD risk factors	Covariates	β	Wald	Odds ratio	95% Confidence Interval	
Coronary heart disease	Role	0.06	0.34	1.06	0.87	1.30
	Gender	0.38	12.68***	1.46	1.18	1.79
	Role*Gender					
Angina	Role					
	Gender	Same as full model				
	Role*Gender					
Heart attack	Role	0.18	3.28	1.20	0.99	1.46
	Gender	0.38	11.72***	1.46	1.17	1.80
	Role*Gender					
Other heart disease	Role	0.10	1.78	1.11	0.95	1.29
	Gender	−0.01	0.01	0.99	0.87	1.14
	Role*Gender					
Stroke	Role	0.19	2.86	1.21	0.97	1.49
	Gender	−0.02	0.03	0.98	0.78	1.23
	Role*Gender					
High blood pressure	Role	0.09	3.13	1.09	0.99	1.21
	Gender	0.12	4.09*	1.13	1.00	1.26
	Role*Gender					
High cholesterol	Role	0.09	2.47	1.09	0.98	1.22
	Gender	0.16	8.43**	1.17	1.05	1.31
	Role*Gender					
Diabetes	Role	0.07	1.03	1.07	0.94	1.23
	Gender	0.21	6.90**	1.23	1.05	1.43
	Role*Gender					
Health behaviors	Covariates					
Currently nonsmoking	Role	−0.12	2.39	0.89	0.75	1.03
	Gender	−0.21	8.46**	0.81	0.71	0.93
	Role*Gender					
Physical activity	Role	−0.09	3.75	0.91	0.84	1.00
	Gender	0.19	15.30***	1.21	1.10	1.33
	Role*Gender					
BMI	Role					
	Gender	Same as full model				
	Role*Gender					
